# My Dream, My Rules: Can Lucid Dreaming Treat Nightmares?

**DOI:** 10.3389/fpsyg.2019.02618

**Published:** 2019-11-26

**Authors:** Tainá Carla Freitas de Macêdo, Glescikelly Herminia Ferreira, Katie Moraes de Almondes, Roumen Kirov, Sérgio Arthuro Mota-Rolim

**Affiliations:** ^1^Department of Psychology, Federal University of Rio Grande do Norte, Natal, Brazil; ^2^Department of Philosophy, Federal University of Pernambuco, Recife, Brazil; ^3^Department of Psychology, Postgraduate Program in Psychobiology, Onofre Lopes University Hospital, Federal University of Rio Grande do Norte, Natal, Brazil; ^4^Institute of Neurobiology, Bulgarian Academy of Sciences, Sofia, Bulgaria; ^5^Brain Institute, Physiology and Behavior Department, Onofre Lopes University Hospital, Federal University of Rio Grande do Norte, Natal, Brazil

**Keywords:** lucid dreaming, nightmare, rapid eye movement sleep, post-traumatic stress disorder, depression, anxiety

## Abstract

Nightmares are defined as repeated occurrences of extremely dysphoric and well-remembered dreams that usually involve subjective threats to survival, security, or physical integrity. Generally, they occur during rapid eye movement sleep (REMS) and lead to awakenings with distress and insufficient overnight sleep. Nightmares may occur spontaneously (idiopathic) or as recurrent nightmares. Recurrent nightmares cause significant distress and impairment in occupational and social functioning, as have been commonly observed in post-traumatic stress disorder, depression and anxiety. By contrast, during lucid dreaming (LD), subjects get insight they are dreaming and may even control the content of their dreams. These features may open a way to help those who suffer from nightmare disorder through re-significations of the dream scene, i.e., knowing that they are dreaming and having control over their dream content. Thus, lucid dreamers might be able to render nightmares normal dreams, thereby assuring a restoring sleep. The aim of the present study is to review the existing literature of the use of LD as an auxiliary tool for treatment of nightmares. We conducted a careful literature search for eligible studies on the use of LD treatment for nightmares. We observed that whereas LD may be a feasible aid in the treatment of patients with nightmares through minimizing their frequency, intensity and psychological distress, the available literature is still scarce and does not provide consistent results. We conclude therefore that more research is clearly warranted for a better estimation of the effective conductance and therapeutic outcome of LD treatment in clinical practice.

## Introduction

According to the International Classification of Sleep Disorders, 3rd Edition (American Academy of Sleep Medicine, 2014), nightmare disorder represents repeated occurrences of extended, extremely dysphoric, and well-remembered dreams that usually involve threats to survival, security, or physical integrity. Nightmares generally occur during rapid eye movement sleep (REMS) and often result in awakening and worsened sleep quality. On awakening from nightmares, subjects rapidly become oriented and alert, but with emotional and physical signs of stress, such as fear, tachycardia, tachypnea, sweating, and daytime impairment in emotion regulation, cognition, and in many social areas of functioning (Levin and Nielsen, 2007; American Academy of Sleep Medicine, 2014; Scarpelli et al., 2019). Nightmares may occur occasionally in almost half of adults, but they may become recurrent, that is, repeated, especially in post-traumatic stress disorder (PTSD) (Hartmann, 1984; Aurora et al., 2010; Morgenthaler et al., 2018), anxiety (Haynes and Mooney, 1975; Levin, 1998; Nielsen et al., 2000; Zadra and Donderi, 2000; Tanskanen et al., 2001) and depression (Germain and Nielsen, 2003; Agargun et al., 2007).

An important etiological distinction made is the difference between idiopathic and posttraumatic nightmares. Idiopathic nightmares are those with unknown etiology and unrelated to other disorders (American Academy of Sleep Medicine, 2014; Robert and Zadra, 2014). Their content is unspecific and includes interpersonal conflict, failure, helplessness, apprehension, being chased, accident, evil force, disaster, and environmental abnormality (Mota-Rolim et al., 2013). According to the “threat simulation theory,” nightmares serve adaptation to stressful events in life (Revonsuo, 2000). However, recent observations point to maladaptive effects of nightmares on sleep and daytime neurobehavioral functions (Levin and Nielsen, 2007; American Academy of Sleep Medicine, 2014; Scarpelli et al., 2019). In contrast, posttraumatic nightmares refer to dreaming disturbances that are part of the stress reaction following exposure to a traumatic event, either during the acute stress response, or over the course of PTSD. Whereas approximately 2–8% of the general population suffers from idiopathic nightmares, nightmares are a core feature of PTSD, with up to 80% of individuals with PTSD reporting disturbing and suicidal dreams with some degree of resemblance to the actual traumatic event (Hasler and Germain, 2009; American Academy of Sleep Medicine, 2014).

Regarding anxiety disorder, it has been found that whereas stress increases frequencies of negative emotions in dreams and nightmares occurrence (Lauer et al., 1987; de Koninck and Brunette, 1991; Köthe and Pietrowsky, 2001), nightmares in turn increase anxiety (Schredl, 2003; Scarpelli et al., 2019). Levin and Fireman (2002) found that in a long run, the reported distress associated with nightmare experience impacted more negatively on quality of life than their frequency did. This finding appears to significantly challenge the “threat simulation theory” (Revonsuo, 2000). It is important to note that whereas nightmare frequency is the number of occurrences of the nightmare, nightmare distress refers to the negative feelings upon awakening following nightmare (Belicki, 1992; Blagrove et al., 2004). In depressed patients, there is a relationship between nightmares and suicides (Agargun et al., 1998; Agargun and Cartwright, 2003; Bernert et al., 2005; Sjöström et al., 2007). The bad feeling soon after awaking from nightmares persists during the rest of the day, being associated with a melancholy and increased suicide risk (Agargun et al., 2007).

The etiology of nightmares is still elusive (Gieselmann et al., 2019). According to the neurocognitive theory, dreams are not mainly generated by the brainstem REMS control, but rather by complex forebrain mechanisms independently of the REMS state (Solms, 2000). According to the impaired fear extinction model (Germain et al., 2008; Nielsen and Levin, 2007), a process of recombining fearful memories with novel and dissociated contexts is continuously activated in nightmare disorder. As stipulated by the affect network dysfunction model (Nielsen and Levin, 2007), individuals high in affect load and affect distress are particularly prone to such impaired fear extinction. In addition, this model is proposed in the trait susceptibility theory of nightmares, which suggests that individuals with frequent nightmares display an increased depth of processing of both negative and positive semantic stimuli (Carr et al., 2016). Finally, all the above factors may contribute to the condensing of recurrent nightmare elements into a nightmare script (Spoormaker, 2008).

Idiopathic nightmares and those related to PTSD, anxiety, depression and other disorders can be treated with lucid dreaming therapy (LDT). Lucid dreams (LD) are those in which the subjects are aware that they are dreaming during the dream, and even may control the oneiric plot (LaBerge, 1980; Mota-Rolim and Araujo, 2013; Baird et al., 2019). This possibility opens a way to help the bearers of nightmares from what is known as re-signification of the dream scene: Being lucid in a nightmare, one can stop fearing the threats by knowing that it is only a dream, and that it could never bring real physical damage. Another tactic would be to face the source of fear, such as monsters, for example (Saint-Denys, 1982), or talk to these monsters in an attempt to find out if they have any specific reason for being there (Tholey, 1988). According to Mota-Rolim and Araujo (2013), individuals can also wake up during the nightmare, try to neutralize it, or even make it enjoyable. Here we would like to answer three basic questions: (1) Is LDT effective for treating nightmares? (2) What are the mechanisms by which LDT works? (3) What are the most used procedures, and the limitations of the LDT?

## Materials and Methods

**FIGURE 1:**
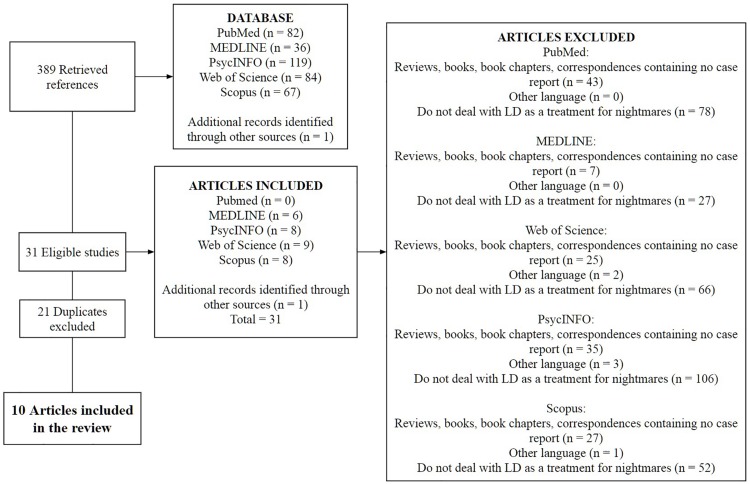
Flowchart showing the screening of the articles that deal with LD as a treatment for recurrent nightmares.

We searched for original research articles in scientific databases, such as PubMed, Medline, PsycINFO, Web of Science, and Scopus using the keywords “lucid dream(s)” or “lucid dreaming” and “nightmare(s)” or “recurrent nightmare.” Our inclusion criteria were: (1) original research articles; (2) written in English; (3) investigated LDT for nightmares. Our exclusion criteria were: (1) original findings replicated in books, book chapters and reviews; (2) purposed on for issues different from clinical use of LDT for recurring nightmares (Figure 1). Data was extracted by three researchers and then reviewed by three (including one that extracted data as well).

## Results

We found 10 original research articles dealing with LDT as a therapeutic approach for nightmares (Table 1). Five case report studies demonstrated beneficial effects of LDT on nightmares and related distress (Halliday, 1982; Brylowski, 1990; Abramovitch, 1995; Tanner, 2004; Been and Garg, 2010). However, case reports cannot prove statistically the beneficial effect of LDT on nightmare frequency, associated distress and worsened sleep quality. Further, several cross-sectional and randomized studies reported for effects of LDT and other psychotherapeutic approaches used to induce LD in order to alleviate basic features of nightmares. Zadra and Pihl (1997) applied long-lasting progressive muscle relaxation and imagery rehearsal therapy (IRT), as a cue for induction of LD in a small sample of recurrent nightmare sufferers. They showed some positive but insignificant effects on nightmare features. Spoormaker et al. (2003) found a positive but also not significant effect of LDT on nightmare frequency and sleep quality. Spoormaker and van den Bout (2006) demonstrated that participants who received individual LDT showed a stronger decrease in nightmare frequency compared to the group that received LDT. Lancee et al. (2010) subjected a larger group of volunteers with self-reported nightmares to IRT, IRT with sleep hygiene and IRT with sleep hygiene and a LD session. They found that application of IRT only was more effective than the other interventions. More recently, Holzinger et al. (2015) subjected participants who suffered from frequent nightmares, and who did not make use of any medication to gestalt therapy (GT) and a combination of GT and LDT. The major results from this randomized study showed that the group that received GT plus LDT had better effects on nightmare features than those subjected to GT only (Table 1).

**TABLE 1:** Summary of the included studies details.

**Citation**	**Sample size and characteristics**	**Study design**	**Intervention(s)**	**Main results or outcomes**

Halliday, 1982	Young, white male, farm worker who suffered vivid recurrent nightmares after a tractor accident.	Case report	The participant was told a story about people who could change their nightmare by introducing a small alteration of some traumatic objects of their dream scene 2–3 times weekly.	The man could change the recurrent nightmare scenario to a pleasant and “lucid” dream by transforming it to neutral and emotionally insignificant object: “the color of a metal shed.”
Brylowski, 1990	A 35-year-old woman who had nightmares associated with borderline personality and depression.	Case report	A 4- to 6-week contact was negotiated for lucid dreaming treatment (LDT), including dream journal, mnemonic induction of lucid dreaming and reading recommendation of a book about lucid dreams during 4–6 weeks.	The techniques used helped the patient to master the negative affect, while the nightmare was still occurring, but with significantly less affective states upon awakening.
Abramovitch (1995)	A 19-year-old woman who suffered an acute nightmare disorder of returning home.	Case report	Home-based (LDT) sessions. Information about the duration and number of sessions was not provided.	The woman was able to modify her nightmare through the lucid dreaming technique.
Zadra and Pihl, 1997	*N* = 5 (recurring nightmare sufferers: 4 women (age range 22–52 years) and 1 man (42-year-old).	Case reports	Two female patients underwent progressive muscle relaxation (PMR) + imagery rehearsal therapy (IRT) + LDT. Three (2 female and 1 male) patients received LDT alone.	One female patient to PMR = IRT + LDT reported no further nightmares at a 4-year follow up. One female patient on LDT tended to decrease her nightmares frequency. Other patients (one female and one male) on LDT reported no further nightmares at 6-month and at 1-year follow-up. The other female patient did not benefit from PMR + IRT + LDT intervention. The effects of both combined and LDT alone can not be assessed statistically due to the study design and low number of reported cases.
Spoormaker et al., 2003	*N* = 8 Anxiety-provoked nightmare sufferers (2 men/6 women; mean age 27.8 years (SD 12.2).	Case reports	All participants received a 1-h Individual, home-based session consisting of (1) lucid dreaming exercises, and (2) of discussing possible constructive solutions for the nightmare.	Nightmare frequency a week decreased up to 60% but not significantly mean (SD) 2.31 (3.56) vs.0.88 (1.13), and sleep quality slightly improved, but also insignificantly due the small sample size used.
Tanner, 2004	A 23 year old woman presenting with a 17 year history of nightmares.	Case report	A combination of relaxation mnemonic procedures to increase lucid dreaming and dream rehearsal upon waking from a nightmare. Four sessions.	Nightmares frequency sharply decreased after four sessions. Further improvement was reported over the next 9 months as additional techniques were introduced and other problems.
Spoormaker and van den Bout, 2006	*N* = 23 (nightmare sufferers; 6 men/17 women; mean age: 28.4 years (SD = 7.3).	Cross-sectional pilot study	12 weeks; Three groups underwent (1) a 2-h individual LDT session (*n* = 8), (2) a 2-h group LDT session (*n* = 8), and (3) waiting list (WL) (*n* = 7) during 12 weeks.	A significant reduction of nightmare frequency for participants who received an individual session (*t*(7) = 4.1, *p* = 0.002). A significant reduction of nightmare frequency was also found in participants who took part in the group session (*t*(7) = 2.6, *p* = 0.02. No significant effects were found in the waiting list group (*t*(6) = 0.6, *p* = 0.30). There were no significant changes between pre intervention and follow-up in sleep quality and overall PTSD symptoms for any of the groups.
Been and Garg, 2010	A 39 year old man with history of depression, PTSD and alcohol dependence. He suffered from insomnia as a result of recurring nightmares. He made use of medications to control anxiety.	Case report	Sixteen days with psychoeducation in LDT based on Wikipedia to realize becoming lucid.	The patient became able to achieve lucidity during his nightmares and then to render them pleasant dreams. The patient did not present any nightmares anymore. His sleep improved and he stopped using medication for anxiety. The patient thinks that the psychoeducation was the main factor for his improvement.
Lancee et al., 2010	*N* = 278: A heterogeneous sample of patients with nightmare disorder (age range: over 33–39 years; 76% female patients).	Randomized controlled trial	Following the exclusion criteria. 67 participants underwent IRT, 75 IRT + sleep hygiene, 71 LDT, and 62 WL. Sessions duration – 6 week. Follow-up measures at weeks 4, 16, and 42.	The IRT alone was more effective than the other intervention conditions over time as measured by the large effect sizes. The effects of LDT alone on the outcome measures were insignificant.
Holzinger et al., 2015	*N* = 40. Patients with recurrent nightmares (10 males/30 females; age range: 20–59 years) who were resistant to medications.	Randomized controlled trial	Thirty-two out of the 40 patients completed the study. One group (*n* = 16) received Gestalt Therapy (GT), while the other group (*n* = 16) received GT + LDT during 10 weeks. Following-up measures at weeks 5 and 10.	Significant reduction of nightmare frequency and improvement of sleep quality in both groups. Dream recall frequency was significantly higher in the group receiving GT + LDT. Compared to the group receiving GT only, the group receiving GT + LDT showed stronger and also significant (*p* ≤ 0.05) effects of the intervention on nightmare frequency and sleep quality at the end of therapy.
				

## Discussion

### What Are the Neurobiological and Psychological Mechanisms That Underlie LDT?

At the neurobiological level, LDT may work by frontal activation, which inhibits the limbic system. During normal REMS, the frontal activity decreases (Maquet et al., 1996); however, during LD the frontal gamma activity (∼40 Hz) increases (Mota-Rolim et al., 2008, 2010; Voss et al., 2009). The frontal region is associated with executive control, attention, rational judgment, working memory, etc. (Hobson, 2009), while the limbic system is related to emotional processes (Peterson et al., 2002). During REMS, there is also an increase in dopamine levels in limbic areas, mainly the nucleus accumbens (Joyce and Meador-Woodruff, 1997; Gottesmann, 2006; McCarley, 2007; Skrzypińska and Szmigielska, 2013). This neurotransmitter pattern and brain areas activity observed in non-lucid REMS are similar to those involved in psychosis (Tort et al., 2005), which may explain the bizarre aspect of dreams (which are analogous to hallucinations), and the lack of rational judgment over this bizarreness (akin the delirious thinking) (Mota et al., 2016). Thus, suppression of the limbic system by the frontal lobe activation during LD could decrease both frequency and intensity of nightmares. Finally, Dresler et al. (2012) observed that the precuneus region is linked to the first-person perspective and agency during LD, which is an important aspect for the treatment of nightmares.

At the psychological level, Rousseau and Belleville (2017) gathers possible mechanisms by which LDT and other similar treatments work, which are: modification of beliefs (Krakow et al., 2000), prevention of avoidance (Pruiksma, 2012), decreased arousal (Davis, 2009), restoration of sleep functions (Germain, 2002), emotional processing (Davis et al., 2007), and sense of mastery (Spoormaker et al., 2003). Change in beliefs can happen both through psychoeducation about the aspects of dreams (Krakow, 2015) and through psychotherapy, focusing on the nightmare theme (Harb et al., 2012). In the case reported by Been and Garg (2010), for example, the patient believes that the psychoeducation was the main factor for his improvement. Avoidance, i.e., trying not to think about the nightmare content or avoiding sleep, is associated with nightmare maintenance (Hansen et al., 2013), and being afraid to fall asleep correlates with higher nightmare frequency (Neylan et al., 1998). Relaxation exercises, as well the sense of mastery itself could help to decrease arousal (Rousseau and Belleville, 2017). Once nightmares are diminished, the subject awakes less, which allows the restoration of sleep functions such as memory consolidation and emotional processing (Germain, 2002). Finally, the belief in control, i.e., the sense of mastery, seems equally important as actually controlling the dream (Spoormaker et al., 2003). Harb et al. (2016) compared the cognitive-behavioral therapy for insomnia (cCBT-I) with IRT + cCBT-I to investigate the potential role of LD as a mechanism of action of IRT in military veterans with PTSD and recurrent nightmares. Before treatment, veterans demonstrated a LD profile characterized by high dream awareness and low dream content control. Following treatment, the control of dream content increased, but lucidity has not changed. This increase in dream content control was related to a reduction in nightmare distress.

Studies show that lucidity is not the main factor to change nightmare content or to reduce nightmare frequency (Spoormaker and van den Bout, 2006). Therefore, a relevant question is: what are the advantages of using LDT over other therapies, e.g., IRT? First, even though lucidity is not the main factor, it does not mean it has no important role. The possibility to achieve lucidity may provide the opportunity to practice self-control and pacific confrontation more directly, which is important to improve the coping ability in the waking state (Brylowski, 1990). According to Lancee et al. (2010), there are two main advantages of LDT over other therapies, especially IRT: (a) once LDT targets the nightmare within the dream, it might be specifically beneficial for people that suffer from non-recurrent nightmares; (b) LDT has more effect on nightmare intensity, because nightmare sufferers achieve a sense of control with the LD technique. Moreover, unlike LDT, IRT might only ameliorate the low intensity nightmares (Lancee et al., 2010). As another advantage, even without lucidity, LDT encourages the attitude of “this is just a nightmare, so there is no real threat.” Although IRT also helps to deal with negative imagery (Krakow and Zadra, 2006), the attitude of “this is just a dream” may play an important role in the modification of belief, decrease of arousal and prevention of avoidance (see Supplementary Material). Despite that, more studies are needed to clarify the mechanisms of therapies that aim to treat nightmares, and to indicate their advantages and disadvantages.

### How Does LDT Work on Practice?

Lucid dreaming therapy for nightmares is a focal modality of psychotherapy. It can happen in a 6-week period (Brylowski, 1990), but can produce effects in a single session (Zadra and Pihl, 1997). The first step is to make it clear that patients have the full capacity to learn how to control their dreams. The therapist guides patients to develop LD induction techniques, and help them to deal with the fear that can follow LD discovery. Once patients feel empowered about their dreams, nightmare frequency might decrease by itself (Spoormaker and van den Bout, 2006). Beyond that, if a nightmare comes up, it will tend to be less distressing, given the sense of mastery that the patient now has. The experience of facing the oneiric threat, i.e., of having a less distressing dream, seems to be essential to the decrease of the remaining nightmares.

Further, a long-term psychotherapy may be initiated, aiming to explore more profoundly the waking life and to elucidate broader questions that even may trigger the nightmares. LDT is a good precedent of a long-term psychotherapy, once it has relatively quick results, which motivates the patient to continue in therapy (Holzinger et al., 2015). Some patients may be skeptic, may have more difficulty to achieve lucidity, or may just have no time to practice frequently at home. In these cases, we recommend using techniques of external sensory stimulation during REMS or substances to induce LD more quickly (Stumbrys et al., 2012; Baird et al., 2019; Mota-Rolim et al., 2019). Nevertheless, some studies demonstrate that even when lucidity is not achieved, exercises of induction facilitates waking up from the nightmare before it becomes too scary (Brylowski, 1990; Tanner, 2004), or changes the oneiric content even without lucidity (Spoormaker and van den Bout, 2006). In these cases, the subjects incorporated elements from the exercises into the dream (Brylowski, 1990; Zadra and Pihl, 1997; Spoormaker et al., 2003). Thus, such exercises helped patients to increase the sense of control over their dreams, consequently, increasing their self-confidence. Moreover, the positive changes in the threatening content are symbolically incorporated to the dreamer’s cognition (Brylowski, 1990).

As said earlier, wake up through lucidity is an option to reduce distress related to nightmares. However, LaBerge and Rheingold (1990) believe that “just wake up” is not as therapeutic as to actually control the content of the dream or the self, once it is a way to run from the nightmare, and not to face it. These authors even suggest that controlling the self is better than controlling the dream content, since in real life, it is not possible to magically change the scenario. Tholey (1988) affirms that when the dream ego looks courageously and openly at hostile dream figures, their appearance often becomes less threatening, as recently supported by Stumbrys and Erlacher (2017) empirical study. On the other hand, when one tries to make a dream figure disappear, it may become even more threatening (Sparrow, 1976). Finally, Tholey (1988) also argue that it is better to conciliate with the dream figure through constructive dialogue than to attack it. Although emotions such as intense fear can trigger lucidity faster (LaBerge and Rheingold, 1990), an unexperienced lucid dreamer is more prone to wake up from the dream than trying to control it, since the excitement caused by the discovery that one is dreaming may cause awakening (Mota-Rolim et al., 2013). Besides, even when subjects are lucid, the fear may not necessarily fade away (Hurd, 2014), thus a “runaway” behavior takes place. Initially, “just wake up” could be a useful weapon until a minimum sense of control is developed; however, it is necessary to practice for the LD scenario does not fade away causing the awakening, which allows the dreamer to explore other possibilities and face their fears.

### What Are the Main Limitations of the LDT?

Halliday (1988) and Zadra (1990) reported case studies in which lucidity was achieved, but without control, and it actually worsened the nightmare. Lucid nightmares are LD with a scary and unpleasant content, in which dreamers have no control over the situation, thus they just “witness” the unfolding of the dream, being unable to deliberately wake up (Hurd, 2009; Schredl and Göritz, 2018; Stumbrys, 2018). Lucid nightmares may be even more terrifying than common nightmares (Halliday, 1988); however, Stumbrys (2018) found that the levels of nightmare vs. lucid nightmare distress do not differ. Sparrow (1991), signifying dreamer’s harrowing experiences with LD, warned about the wholesale advertising of LD, since lucid nightmares frequency is associated not only to nightmare frequency, but also to LD frequency (Stumbrys, 2018). This makes patients with nightmares very vulnerable to lucid nightmares in a LDT. Therefore, some care is needed when a LDT is initiated. Fortunately, community support is helpful in reducing lucid nightmares (Hurd, 2006). Besides, it was found that dopamine agonists are useful in reducing lucid nightmares frequency (McLaughlin et al., 2015a, b, 2016); however, these studies comprise only a few cases in a very special population, which limits the generalizability of the findings. Finally, one main issue in LDT is to induce LD, which is usually difficult for most of people (Stumbrys et al., 2012), who experience LD rarely (Mota-Rolim et al., 2013). However, Dodet et al. (2015) and Rak et al. (2015) observed that narcoleptic patients report more LD than the rest of population, and that some of these patients even learned to use LD to change their recurrent nightmares. These authors suggest that the experience of these patients with LD could help other narcoleptics who suffer from frequent nightmares.

## Conclusion

Lucid dreaming therapy may be efficient for treating nightmares, and even when lucidity is not achieved, the induction exercises assisted patients by helping them develop a critical thinking over dream content. Although induction of LD may be a feasible aid in the treatment of patients with nightmares through minimizing their frequency, intensity and psychological distress, the available literature is still scarce and does not provide consistent results. Furthermore, the samples size are limited, which precludes more significant comparisons. Therefore, more research is clearly warranted for a better estimation of the effective conductance and therapeutic outcome of LD techniques in clinical practice.

## Author Contributions

TM and RK conducted the literature search, selected the eligible studies, and drafted the manuscript. GF drafted the manuscript. KA selected the eligible studies and drafted the manuscript. SM-R designed the manuscript, conducted the literature search, selected the eligible studies, and drafted the manuscript. All authors worked over the first draft of the manuscript and approved the final version.

## Conflict of Interest

The authors declare that the research was conducted in the absence of any commercial or financial relationships that could be construed as a potential conflict of interest.

## References

[B1] Abramovitch H. (1995). The nightmare of returning home: a case of acute onset nightmare disorder treated by lucid dreaming. *Isr. J. Psychiatry Relat. Sci.* 32 140–145. 7558759

[B2] Agargun M. Y. Besiroglu L. Cilli A. S. Gulec M. Aydin A. Inci R. (2007). Nightmares, suicide attempts, and melancholic features in patients with unipolar major depression. *J. Affect Disord.* 98 267–270. 10.1016/j.jad.2006.08.005 16938351

[B3] Agargun M. Y. Cartwright R. (2003). REM sleep, dream variables and suicidality in depressed patients. *Psychiatry Res.* 119 33–39. 10.1016/s0165-1781(03)00111-2 12860358

[B4] Agargun M. Y. Cilli A. S. Kara H. Tarhan N. Kincir F. Oz H. (1998). Repetitive and frightening dreams and suicidal behavior in patients with major depression. *Compr. Psychiatry* 39 198–202. 10.1016/s0010-440x(98)90060-8 9675503

[B5] American Academy of Sleep Medicine (2014). *International Classification of Sleep Disorders*, 3rd Edn Darien, IL: American Academy of Sleep Medicine.

[B6] Aurora R. N. Zak R. S. Auerbach S. H. Casey K. R. Chowdhuri S. Karippot A. (2010). Best practice guide for the treatment of nightmare disorder in adults. *J. Clin. Sleep Med.* 6 389–401. 20726290 PMC2919672

[B7] Baird B. Mota-Rolim S. A. Dresler M. (2019). The cognitive neuroscience of lucid dreaming. *Neurosci. Biobehav. Rev.* 100 305–323. 10.1016/j.neubiorev.2019.03.008 30880167 PMC6451677

[B8] Been G. Garg V. (2010). Nightmares in the context of PTSD treated with psychoeducation regarding lucid dreaming. *Aust. N. Z. J. Psychiatry* 44:583. 10.1080/00048671003614213 20482419

[B9] Belicki K. (1992). Nightmare frequency versus nightmare distress: relations to psychopathology and cognitive style. *J. Abnorm. Psychol.* 101 592–597. 10.1037/0021-843x.101.3.592 1500619

[B10] Bernert R. A. Joiner T. E. Jr. Cukrowicz K. C. Schmidt N. B. Krakow B. (2005). Suicidality and sleep disturbances. *Sleep* 28 1135–1141. 10.1093/sleep/28.9.1135 16268383

[B11] Blagrove M. Farmer L. Williams E. (2004). The relationship of nightmare frequency and nightmare distress to well-being. *J. Sleep Res.* 13 129–136. 10.1111/j.1365-2869.2004.00394.x 15175092

[B12] Brylowski A. (1990). Nightmares in crisis: clinical applications of lucid dreaming techniques. *Psychiatr. J. Univ. Ott.* 15 79–84. 2374792

[B13] Carr M. Blanchette-Carrière C. Marquis L. P. Ting C. Nielsen T. (2016). Nightmare sufferers show atypical emotional semantic breadth and prolonged REM sleep-dependent emotional priming. *Sleep Med Rev.* 20 80–87. 10.1016/j.sleep.2015.11.013 27318230

[B14] Davis J. L. (2009). *Treating Post-Trauma Nightmares: A Cognitive Behavioral Approach.* New York, NY: Springer Publishing Co.

[B15] Davis J. L. Byrd P. Rhudy J. L. Wright D. C. (2007). Characteristics of chronic nightmares in a trauma-exposed treatment-seeking sample. *Dreaming* 17 187–198. 10.1037/1053-0797.17.4.187

[B16] de Koninck J. Brunette R. (1991). Presleep suggestion related to a phobic object: successful manipulation of reported dream affect. *J. Gen. Psychol.* 118 185–200. 10.1080/00221309.1991.9917780 1757780

[B17] Saint-Denys H. de. (1982). “*Dreams and the Means of Directing Them*,” in *From the French Les Reves et Les Moyens de Les Diriger (1867). Paris: Amyat*, ed. Schatzman M. Trans Fry N. (London: Duckworth).

[B18] Dodet P. Chavez M. Leu-Semenescu S. Golmard J. L. Arnulf I. (2015). Lucid dreaming in narcolepsy. *Sleep* 38 487–497. 10.5665/sleep.4516 25348131 PMC4335518

[B19] Dresler M. Wehrle R. Spoormaker V. I. Koch S. P. Holsboer F. Steiger A. (2012). Neural correlates of dream lucidity obtained from contrasting lucid versus non-lucid REM sleep: a combined EEG/fMRI case study. *Sleep* 35 1017–1020. 10.5665/sleep.1974 22754049 PMC3369221

[B20] Germain A. (2002). *Sleep Pathophysiology and Cognitive-Behavioral Treatment of Posttraumatic and Idiopathic Nightmares.* Ann Arbor, MI: PQIL.

[B21] Germain A. Buysse D. J. Nofzinger E. A. (2008). Sleep – specific mechanisms underlying posttraumatic stress disorder: integrative review and neurobiological hypotheses. *Sleep Med Rev.* 12 185–195. 10.1016/j.smrv.2007.09.003 17997114 PMC2490669

[B22] Germain A. Nielsen T. A. (2003). Sleep pathophysiology in posttraumatic stress disorder and idiopathic nightmare sufferers. *Biol. Psychiatry.* 54 1092–1098. 10.1016/s0006-3223(03)00071-4 14625152

[B23] Gieselmann A. Ait Aoudia M. Carr M. Germain A. Gorzka R. Holzinger B. (2019). Aetiology and treatment of nightmare disorder: state of the art and future perspectives. *J. Sleep Res.* 28:e12820. 10.1111/jsr.12820 30697860 PMC6850667

[B24] Gottesmann C. (2006). The dream sleeping stage: a new neurobiological model schizophrenia? *Neuroscience* 140 1105–1115. 10.1016/j.neuroscience.2006.02.082 16650940

[B25] Halliday G. (1982). Direct alteration of a traumatic nightmare. *Percep. Mot. Skills* 54 413–414. 10.2466/pms.1982.54.2.413 7079069

[B26] Halliday G. (1988). “Lucid dreaming: use in nightmares and sleep-wake confusion,” In *Conscious Mind, Sleeping Brain*, (Eds.) Gackenbach J. LaBerge S. (New York, NY: Plenum Press), 306.

[B27] Hansen K. Höfling V. Kröner-Borowik T. Stangier U. Steil R. (2013). Efficacy of psychological interventions aiming to reduce chronic nightmares: a meta-analysis. *Clin. Psychol. Rev.* 33 146–155. 10.1016/j.cpr.2012.10.012 23186732

[B28] Harb G. C. Brownlow J. A. Ross R. J. (2016). Posttraumatic nightmares and imagery rehearsal: the possible role of lucid dreaming. *Dreaming* 26 238–249. 10.1037/drm0000030

[B29] Harb G. C. Thompson R. Ross R. J. Cook J. M. (2012). Combat-related PTSD nightmares and imagery rehearsal: nightmare characteristics and relation to treatment outcome. *J. Trauma Stress* 25 511–518. 10.1002/jts.21748 23047646

[B30] Hartmann E. (1984). *The Nightmare: The Psychology and Biology of Terrifying Dreams.* New York, NY: Basic Books, 294.

[B31] Hasler B. Germain A. (2009). Correlates and treatments of nightmares in adults. *Sleep Med. Clin.* 4 507–517. 10.1016/j.jsmc.2009.07.012 20161576 PMC2806673

[B32] Haynes S. N. Mooney D. K. (1975). Nightmares: etiological, theoretical, and behavioral treatment considerations. *Psychol. Rec.* 25 225–236. 10.1007/BF03394308

[B33] Hobson A. (2009). The neurobiology of consciousness: lucid dreaming wakes up. *Int. J. Dream Res.* 2 41–44. 10.11588/ijodr.2009.2.403

[B34] Holzinger B. Klösch G. Saletu B. (2015). Studies with lucid dreaming as add-on therapy to gestalt therapy. *Acta Neurol. Scand.* 131 355–363. 10.1111/ane.12362 25639732

[B35] Hurd R. (2006). “Lucid dreaming: new perspectives of consciousness in sleep,” in *Proceedings of the Presentation at “The Transformative Power of Dream Studies” Panel, the Annual Conference of the International Association for the Study of Dreams*, (Bridgewater, MA).

[B36] Hurd R. (2009). “Lucid nightmares: the dark side of self-awareness in dreams,” in *Proceedings of the Annual Conference of the International Association for the Study of Dreams*, (Chicago, IL).

[B37] Hurd R. (2014). ““Unearthing the Paleolithic Mind in Lucid Dreams,” in *Lucid Dreaming: New Perspectives on Consciousness*, eds Hurd R. Bulkeley K. (Santa Barbara, CA: ABC-CLIO), 277–324.

[B38] Joyce J. N. Meador-Woodruff J. H. (1997). Linking the family of D2 receptors to neuronal circuits in human brain: insights into schizophrenia. *Neuropsychopharmacology* 16 375–384. 10.1016/s0893-133x(96)00276-x 9165493

[B39] Köthe M. Pietrowsky R. (2001). Behavioral effects of nightmares and their correlations to personality patterns. *Dreaming* 11 43–52. 10.1023/A:1009468517557

[B40] Krakow B. (2015). “Nightmare therapy: emerging concepts from sleep medicine,” in *Dream Research: Contributions to Clinical Practice*, ed. Glucksman M. K. M. (New York, NY: Routledge/Taylor & Francis Group), 149–160.

[B41] Krakow B. Hollifield M. Schrader R. Koss M. Tandberg D. Lauriello J. (2000). A controlled study of imagery rehearsal for chronic nightmares in sexual assault survivors with PTSD: a preliminary report. *J. Trauma Stress* 13 589–609. 10.1023/a:1007854015481 11109233

[B42] Krakow B. Zadra A. (2006). Clinical management of chronic nightmares: imagery rehearsal therapy. *Behav. Sleep Med.* 4 45–70. 10.1207/s15402010bsm0401_4 16390284

[B43] LaBerge S. (1980). Lucid dreaming as a learnable skill: a case study. *Percept. Motor Skills* 51 1039–1042. 10.2466/pms.1980.51.3f.1039

[B44] LaBerge S. Rheingold H. (1990). *Exploring the World of Lucid Dreaming.* New York, NY: Ballantine Books.

[B45] Lancee J. van den Bout J. Spoormaker V. I. (2010). Expanding self-help imagery rehearsal therapy for nightmares with sleep hygiene and lucid dreaming: a waiting-list controlled trial. *Int. J. Dream Res.* 3 111–120. 10.11588/ijodr.2010.2.6128

[B46] Lauer C. Riemann D. Lund R. Berger M. (1987). Shortened REM latency: a consequence of psychological strain? *Psychophysiology* 24 263–271. 10.1111/j.1469-8986.1987.tb00293.x 3602281

[B47] Levin R. (1998). Nightmares and schizotypy. *Psychiatry* 61 206–216. 10.1080/00332747.1998.11024832 9823030

[B48] Levin R. Fireman G. (2002). Nightmare prevalence, nightmare distress, and self-reported psychological disturbance. *Sleep* 25 205–212. 11902430

[B49] Levin R. Nielsen T. A. (2007). Disturbed dreaming, posttraumatic stress disorder, and affect distress: a review and neurocognitive model. *Psychol. Bull.* 133 482–528. 10.1037/0033-2909.133.3.482 17469988

[B50] Maquet P. Péters J.-M. Aerts J. Delfiore G. Degueldre C. Luxen A. (1996). Functional neuroanatomy of human rapid-eye-movement sleep and dreaming. *Nature* 383 163–166. 10.1038/383163a0 8774879

[B51] McCarley R. W. (2007). Neurobiology of REM and NREM sleep. *Sleep Med.* 8 302–330. 10.1016/j.sleep.2007.03.005 17468046

[B52] McLaughlin T. Blum K. Oscar-Berman M. Febo M. Agan G. Fratantonio J. L. (2015a). Putative dopamine agonist (KB220Z) attenuates lucid nightmares in PTSD patients: role of enhanced brain reward functional connectivity and homeostasis redeeming joy. *J. Behav. Addict.* 4 106–115. 10.1556/2006.4.2015.008 26132915 PMC4500891

[B53] McLaughlin T. Blum K. Oscar-Berman M. Febo M. Demetrovics Z. Agan G. (2015b). Using the neuroadaptagen KB200z^TM^ to ameliorate terrifying, lucid nightmares in RDS patients: the role of enhanced, brain-reward, functional connectivity and dopaminergic homeostasis. *J. Reward Defic. Syndr.* 1 24–35. 10.17756/jrds.2015-006 26065033 PMC4459746

[B54] McLaughlin T. Febo M. Badgaiyan R. D. Barh D. Dushaj K. Braverman E. R. (2016). KB220Z^TM^ a pro-dopamine regulator associated with the protracted, alleviation of terrifying lucid dreams. Can we infer neuroplasticity-induced changes in the reward circuit? *J. Reward Defic. Syndr. Addict. Sci.* 2 3–13. 10.17756/jrdsas.2016-022 28210713 PMC5308138

[B55] Morgenthaler T. I. Auerbach S. Casey K. R. Kristo D. Maganti R. (2018). Position paper for the treatment of nightmare disorder in adults: an american academy of sleep medicine position paper. *J. Clin. Sleep Med.* 14 1041–1055. 10.5664/jcsm.7178 29852917 PMC5991964

[B56] Mota N. B. Resende A. Mota-Rolim S. A. Copelli M. Ribeiro S. (2016). Psychosis and the control of lucid dreaming. *Front. Psychol.* 7:294. 10.3389/fpsyg.2016.00294 27014118 PMC4783408

[B57] Mota-Rolim S. Pantoja A. Pinheiro R. Camilo A. Barbosa T. Hazboun I. (2008). “Lucid dream: sleep electroencephalographic features and behavioral induction methods,” in *First Congress IBRO/LARC of Neurosciences for Latin America, Caribbean and Iberian Peninsula*, (Búzios).

[B58] Mota-Rolim S. A. Araujo J. F. (2013). Neurobiology and clinical implications of lucid dreaming. *Med. Hypotheses* 81 751–756. 10.1016/j.mehy.2013.04.049 23838126

[B59] Mota-Rolim S. A. Erlacher D. Tort A. B. L. Araujo J. F. Ribeiro S. (2010). Different kinds of subjective experience during lucid dreaming may have different neural substrates. *Int. J. Dream Res.* 3 33–35. 10.11588/ijodr.2010.1.596

[B60] Mota-Rolim S. A. Pavlou A. Nascimento G. Araujo J. F. Ribeiro S. (2019). Portable devices to induce lucid dreams – are they reliable? *Front. Neurosci.* 13:428 10.3389/fnins.2019.00428 PMC6517539 31133778

[B61] Mota-Rolim S. A. Targino Z. H. Souza B. C. Blanco W. Araujo J. F. Ribeiro S. (2013). Dream characteristics in a Brazilian sample: an online survey focusing on lucid dreaming. *Front. Hum. Neurosci.* 7:836. 10.3389/fnhum.2013.00836 24368900 PMC3857923

[B62] Neylan T. C. Marmar C. R. Metzler T. J. Weiss D. S. Zatzick D. F. Delucchi K. L. (1998). Sleep disturbances in the vietnam generation: findings from a nationally representative sample of male vietnam veterans. *Am. J. Psychiatry* 155 929–933. 10.1176/ajp.155.7.929 9659859

[B63] Nielsen T. Levin R. (2007). Nightmares: a new neurocognitive model. *Sleep Med. Rev.* 11 295–310. 10.1016/j.smrv.2007.03.004 17498981

[B64] Nielsen T. A. LaBerge L. Paquet J. Tremblay R. E. Vitaro F. Montplaisir J. (2000). Development of disturbing dreams during adolescence and their relation to anxiety symptoms. *Sleep* 23 727–736. 11007439

[B65] Peterson N. D. J. Henke P. G. Hayes Z. (2002). Limbic system function and dream content in university students. *J. Neuropsychiatry Clin. Neurosci.* 14 283–288. 10.1176/appi.neuropsych.14.3.283 12154152

[B66] Pruiksma K. M. E. (2012). *A Randomized Controlled Trial of Exposure, Relaxation, and Rescripting Therapy and Relaxation Training for Chronic Nightmares in Trauma-Exposed Persons: Findings at one Week Posttreatment.* Tulsa, OK: University of Tulsa .

[B67] Rak M. Beitinger P. Steiger A. Schredl M. Dresler M. (2015). Increased lucid dreaming frequency in narcolepsy. *Sleep* 38 787–792. 10.5665/sleep.4676 25325481 PMC4402667

[B68] Revonsuo A. (2000). The reinterpretation of dreams: an evolutionary hypothesis of the function of dreaming. *Behav. Brain Sci.* 23 877–901. 10.1017/s0140525x00004015 11515147

[B69] Robert G. Zadra A. (2014). Thematic and content analysis of idiopathic nightmares and bad dreams. *Sleep* 37 409–417. 10.5665/sleep.3426 24497669 PMC3900621

[B70] Rousseau A. Belleville G. (2017). The mechanisms of action underlying the efficacy of psychological nightmare treatments: a systematic review and thematic analysis of discussed hypotheses. *Sleep Med. Rev.* 39 122–133. 10.1016/j.smrv.2017.08.004 29056416

[B71] Scarpelli S. Bartolacci C. D’Atri A. Gorgoni M. De Gennaro L. (2019). The functional role of dreaming in emotional processes. *Front. Psychol.* 10:459. 10.3389/fpsyg.2019.00459 30930809 PMC6428732

[B72] Schredl M. (2003). Effects of state and trait factors on nightmare frequency. *Eur. Arch. Psychiatry Clin. Neurosci.* 253 241–247. 10.1007/s00406-003-0438-1 14504993

[B73] Schredl M. Göritz A. S. (2018). Nightmare themes: an online study of most recent nightmares and childhood nightmares. *J. Clin. Sleep Med.* 14 465–471. 10.5664/jcsm.7002 29458691 PMC5837849

[B74] Sjöström N. Wærn M. Hetta J. (2007). Nightmares and sleep disturbances in relation to suicidality in suicide attempters. *Sleep* 30 91–95. 10.1093/sleep/30.1.91 17310869

[B75] Skrzypińska D. Szmigielska B. (2013). What links schizophrenia and dreaming? Common phenomenological and neurobiological features of schizophrenia and REM sleep. *Arch. Psychiatry Psychother.* 2 29–35. 10.12740/app/18443

[B76] Solms M. (2000). Dreaming and REM sleep are controlled by different brain mechanisms. *Behav. Brain Sci.* 23 843–850. 10.1017/s0140525x00003988 11515144

[B77] Sparrow G. S. (1976). *Lucid Dreaming: Dawning of the Clear Light.* Virginia Beach, VA: A. R. E. Press, 33.

[B78] Sparrow G. S. (1991). Letter from Scott Sparrow. *Lucidity* 10 421–424.

[B79] Spoormaker V. I. (2008). A cognitive model of recurrent nightmares. *Int. J. Dream Res.* 1 15–22. 11515147

[B80] Spoormaker V. I. Van De Bout J. Meijer E. J. G. (2003). Lucid dreaming treatment for nightmares: a series of cases. *Dreaming* 13 181–186. 10.1037/1053-0797.13.3.181

[B81] Spoormaker V. I. van den Bout J. (2006). Lucid dreaming treatment for nightmares: a pilot study. *Psychother. Psychosom.* 75 389–394. 10.1159/000095446 17053341

[B82] Stumbrys T. (2018). Lucid nightmares: a survey of their frequency, features, and factors in lucid dreamers. *Dreaming* 28 193–204. 10.1037/drm0000090

[B83] Stumbrys T. Erlacher D. (2017). Inner ghosts: encounters with threatening dream characters in lucid dreams. *Dreaming* 27 40–48. 10.1037/drm0000043

[B84] Stumbrys T. Erlacher D. Schadlich M. Schredl M. (2012). Induction of lucid dreams: a systematic review of evidence. *Conscious. Cogn.* 21 1456–1475. 10.1016/j.concog.2012.07.003 22841958

[B85] Tanner B. (2004). Multimodal behavioral treatment of nonrepetitive, treatment-resistant nightmares: a case report. *Percept. Mot. Skills* 99 1139–1146. 10.2466/pms.99.3f.1139-1146. 15739837

[B86] Tanskanen A. Tuomilehto J. Viinamaki H. Vartiainen E. Lehtonen J. Puska P. (2001). Nightmares as predictors of suicide. *Sleep* 24 845–848. 11683487

[B87] Tholey P. (1988). “A model for lucidity training as a means of self-healing and psychological growth,” in *Conscious Mind, Sleeping Brain: Perspectives on Lucid Dreaming*, eds Gackenbach J. LaBerge S. (New York, NY: Plenum), 265.

[B88] Tort A. B. Dall’Igna O. P. de Oliveira R. V. Mantese C. E. Fett P. Gomes M. W. (2005). Atypical antipsychotic profile of flunarizine in animal models. *Psychopharmacology* 177 344–348. 10.1007/s00213-004-1955-y 15290004

[B89] Voss U. Holzmann R. Tuin I. Hobson J. A. (2009). Lucid dreaming: a state of consciousness with features of both waking and non-lucid dreaming. *Sleep* 32 1191–1200. 10.1093/sleep/32.9.1191 19750924 PMC2737577

[B90] Zadra A. Donderi D. C. (2000). Nightmares and bad dreams: their prevalence and relationship to well-being. *J. Abnorm. Psychol.* 109 273–281. 10.1037//0021-843x.109.2.273 10895565

[B91] Zadra A. L. (1990). “Lucid dreaming, dream control, and the treatment of nightmares,” in *Proceedings of the Paper Presented at the Seventh Annual Conference of the Association for the Study of Dreams*, (Chicago).

[B92] Zadra A. L. Pihl R. O. (1997). Lucid dreaming as a treatment for recurrent nightmares. *Psychother. Psychosom.* 66 50–55. 10.1159/000289106 8996716

