# Supplementary material for: My Dream, My Rules: Can Lucid Dreaming Treat Nightmares?
Source: Front Psychol. 2019 Nov 26;10:2618. doi: 10.3389/fpsyg.2019.02618 (PMC6902039; doi:10.3389/fpsyg.2019.02618)
Supplement: Supplementary file 1 [file Table_1.doc]

**Major differences between lucid dreaming treatment (LDT) and imagery rehearsal therapy (IRT) in treatment of nightmare disorder: Tentative advantages and disadvantages**

Compared to IRT, LTD takes a notably different approach focused on enabling individuals to influence their dreams directly while dreaming (Holzinger, 2014). A lucid dream (LD) is characterized by an awareness of orientation, that is, of being in the process of dreaming; it is often also accompanied by an awareness of being able to make decisions and therefore to take charge of or responsibility for the dream plot. This may lead to the ability to act volitionally. For example, subjects are able to alter or even may control their dream's content or to simply wake oneself up (Baird et al., 2019). In practice, patients are invited to go to bed with the explicit intent to improve their dream recall by writing down their dreams directly upon awakening. In addition, adopting the conviction that one can realize when one is dreaming was found to increase the frequency of LD. Checking whether or not one has become lucid can be encouraged during wakefulness with reality testing methods that are repeated frequently: ‘‘Is this really happening or am I dreaming?’’, ‘‘When I look at something twice, does it still look the same (e.g. Does the clock still indicate the same time or not? Is the book title still the same or is it not)?’’ See for reviews (Holzinger, 2014; Tholey, 1988).

The IRT approach to treat nightmares is underpinned by the best available evidence and therefore recommended as level A treatment for nightmare disorder by the Oxford Centre for Evidence Based Medicine (Cranston et al., 2011), as well as by the AASM (Aurora et al., 2010). With IRT, patients are instructed to rescript the story of the nightmare during wakefulness. The original instruction was to: “Change the nightmare regardless of one’s wish” (Kellner et al., 1992), which offered no specific targeting of the nightmare's ending or any other specified component of the disturbing dream, whereas others have proposed to change the nightmare to possess a “triumphant” (Marks, 1978) - “satisfactory” or “more neutral or even pleasant”(Thünker and Pietrowsky, 2012). This rescripted nightmare, which no longer appears to be a nightmare is then rehearsed in the patients’ imagination a couple of times each day. In their meta-analysis, Hansen et al., (2013) reported large pre–post within-group large effect sizes for IRT for reducing of nightmare frequency (Augedal et al., 2013). Compared to other meta-analyses, neither adverse effects, nor symptom substitutions were reported in patients being treated with IRT.

In conclusion, both LDT and IRT might represent useful psychotherapeutic approaches in treatment of nightmare disorder.

Grounding on the literature sources so far available, it seems that IRT may have more advantages than LDT. **1.** IRT does not affect sleep dreaming conscious states directly but as a cue for induction of LD, and was thus shown to exert lower than LDT side effects, respectively. However, this might be due to the higher number of trials using IRT for nightmare disorder than the number of studies using LDT. **2.** Next, the effectiveness of LDT for nightmare disorder is still less well understood. This is mostly because its effectiveness and safety for treatment of nightmares have been explored frequently in case report studies, in studies that have used small sample sizes and in randomized studies that have applied a combination of LDT and other techniques (Please refer to Table 1 in the manuscript).

The advantages of LDT over IRT might be the following. **1.** LDT targets sleep dreaming state more directly than IRT, thus proposing a greater than the reported so far beneficial effects on nightmare disorder. **2.** Compared to IRT, LDT allows patients to be aware of their dreaming state and opens a possibility to control the oneiric plot. **3.** While the changes in brain neurophysiology following IRT for nightmares are largely unknown, those following LD are better recognized (Holzinger et al., 2006; Voss et al., 2009; Baird et al., 2019). This opens a new avenue for using LDT as a reliable and neurophysiologically proved approach for coping with nightmare disorder.

**References**

Augedal, A.W., Hansen, K.S., Kronhaug, C.R., Harvey, A.G., and Pallesen, S. (2013). Randomized controlled trials of psychological and pharmacological treatments for nightmares: A meta-analysis. Sleep Med Rev. 17, 143–152. https://doi.org/10.1016/j.

smrv.2012.06.001.

Aurora, R.N., Zak, R.S., Auerbach, S.H., Casey, K R., Chowdhuri, S., and Karippot, A. (2010). Standards of practice committee: American Academy of Sleep Medicine Best practice guide for the treatment of nightmare disorder in adults. Journal Clin Sleep Med. 38, 9–401. https://www.ncbi.nlm.nih.gov.

Baird, B., Mota-Rolim, S.A., Dresler, M. (2019). The cognitive neuroscience of lucid dreaming. Neurosci. Biobehav. Rev. 100, 305-323. doi: 10.1016/j.neubiorev.2019.03.008.

Cranston, C.C., Davis, J L., Rhudy, J.L. and Favorite, T.K. (2011). Replication and expansion of “best practice guide for the treatment of nightmare disorder in adults”. Journal Clin Sleep Med. 7, 549–553. doi: 10.5664/JCSM.1330.

Hansen, K., Höfling, V., Kröner-Borowik, T., Stangier, U., and Steil, R. (2013).

Efficacy of psychological interventions aiming to reduce chronic nightmares: A meta-nalysis. Clin Psychol Rev. 33, 146–155. https://doi.org/10.1016/j.cpr.2012.10.012.

Holzinger, B., LaBerge, S., & Levitan, L. (2006). Psychophysiological correlates of lucid dreaming. Dreaming, 16(2), 88–95. doi:10.1037/1053-0797.16.2.88.

Holzinger, B. (2014). Lucid dreaming in psychotherapy. In R. Hurd, & K. Bukeley (Eds.), Lucid dreaming: New perspectives on consciousness in sleep. Westport: Praeger, pp. 37-62. ISBN: 13: 978-1440829475.

Kellner, R., Neidhardt, J., Krakow, B., and Pathak, D. (1992). Changes in chronic nightmares after one session of desensitization or rehearsal instructions. Am J Psychiatry 149, 659–663. doi: 10.1176/ajp.149.5.659.

Marks, I. (1978). Rehearsal relief of a nightmare. British J Psychiatry 133, 461–465. <https://doi.org/10.1192/bjp.133.5.461>

Tholey, P. (1988). A model for lucidity training as a means of self-healing and psychological growth. In: Gackenbach, J., and LaBerge, S. (Eds.), Conscious mind, sleeping brain: perspectives on lucid dreaming. New York: Plenum. 265. doi: 10.1007/978-1-4757-0423-5.

Thünker, J., and Pietrowsky, R. (2012). Effectiveness of a manualized imagery rehearsal therapy for patients suffering from nightmare disorders with and without a comorbidity of depression or PTSD. Behav Res Therapy 50, 558–564. <https://doi.org/10.1016/j. brat.2012.05.006>.

Voss, U., Holzmann, R., Tuin, I., Hobson, J.A. (2009). Lucid Dreaming: A State of Consciousness with Features of both Waking and Non-Lucid Dreaming. Sleep, 32 (9), 1191-1200. doi: 10.1093/sleep/32.9.1191.
