# Supplementary material for: My Dream, My Rules: Can Lucid Dreaming Treat Nightmares?
Source: Front Psychol. 2019 Nov 26;10:2618. doi: 10.3389/fpsyg.2019.02618 (PMC6902039; doi:10.3389/fpsyg.2019.02618)
Supplement: Supplementary file 2 [file Table_2.doc]

**Practice agenda of a reliable technique for applying lucid dreaming in treatment of nightmares: The Combined Technique (Tholey, 1983):**

Our research has shown that the reflection technique is an effective technique for inducing lucid dreams. This was especially evident in the case of subjects who had never previously experienced a lucid dream. This technique includes a daily interview but is applied at the point of falling in sleep. In the following, we will describe a combined technique which primarily stresses training in the development of a critical reflective frame of mind, but which also contains elements of intention and suggestion.

**(1)** The subject should ask him-/ her-self the critical question ("am I dreaming or not?") at least five to ten times a day.

**(2)** At the same time, the subject should try to imagine intensely that he/she is in a dream state, that is, that everything he/she perceives, including his/her own body, is merely a dream.

**(3)** While asking patient the critical question the subject should concentrate not only on contemporary occurrences, but also on events that have already taken place. Does he/she come upon something unusual, or does he/she suffer from lapses of memory? A minute suffices to answer the question.

**(4)** The subject should ask him/herself the critical question as a rule in all situations that are characteristic for dreams, that is, whenever something surprising or improbable occurs or whenever he/she experiences powerful emotions.

**(5)** It is especially helpful in learning how to dream lucidly if the subject has dreams with a recurrent content. For example, if he/she frequently has feelings of fear or often sees dogs in their dreams, then he/she should ask themselves the critical question concerning his/her state of consciousness whenever he/she finds himself in threatening situations or sees a dog in the daytime.

**(6)** If the subject often has dream experiences that never or very seldom occur in a waking state, such as floating or flying, then he/she should, while awake, try intensely to imagine that he/she is having such an experience, telling him-/ her-self all the while that he is dreaming.

**(7)** If the subject has difficulty recalling his normal dreams, he/she should employ methods for improving dream recollection such as are described in recent literature on dreaming, e.g., a dream journal. In most cases, however, practice in attaining the critical-reflective frame of mind will improve the subject's ability to recall his dreams.

**(8)** The subject should go to sleep thinking that he/she is going to attain awareness of dreaming while in this state. Any conscious effort of will must be avoided while thinking their thought. This method is especially effective when the subject has just awakened in the early morning hours and has the feeling that he is about to fall asleep again.

**(9)** The subject should resolve to carry out a particular action while dreaming. This may prevent the subject of wasting time in the dream trying to decide what to do and may avoid or retard loss of lucidity during the dream.

Based on our findings we conjecture that whoever consistently follows the advice given can learn to dream lucidly. Subjects who have never previously experienced a lucid dream will have the first one after a median time of 4 to 5 weeks. Under the most favorable circumstances, the subject will experience his/her first lucid dream during the very first night, under unfavorable circumstances only after several months.

**Some more popular and easily understood steps that may be implemented at the point of falling in sleep:**

**1.** Make your bedroom hospitable to dreaming.

**2.** Keep a dream journal.

**3.** Recognize your dream signs.

**4.** Perform reality checks.

**5.** Use the mild imagination lucid dreaming technique.

**6.** Try going back to sleep upon awakening.

**7.** Use the wake back to bed technique.

This practice in attaining a critical-reflective frame of mind is only necessary in the beginning phase, which may last a number of months. Later on, lucid dreams will frequently occur when if the subject has not asked him/herself the critical question during the day. The frequency of lucid dreams then depends to a large extent on the will of the subject. Most subjects who consistently follow the above advice experience at least one lucid dream every night.

**Reference**

Tholey, P. (1983). Techniques for inducing and maintaining lucid dreams. Percept Mot Skills 57, 79-90. <https://doi.org/10.2466/pms.1983.57.1.79>.
